# Whole-genome sequence of *Ensifer aridi* strain RCAM05007 isolated from *Cyamopsis tetragonoloba* (L.) Taub. root nodule

**DOI:** 10.1128/mra.01012-24

**Published:** 2024-11-22

**Authors:** Polina Guro, Irina Kuznetsova, Anna Sazanova, Andrey Belimov, Denis Karlov, Vera Safronova

**Affiliations:** 1 All-Russia Research Institute for Agricultural Microbiology (ARRIAM), St. Petersburg, Russia; The University of Arizona, Tucson, Arizona, USA

**Keywords:** guar, *Ensifer*

## Abstract

We present the genome sequence of *Ensifer aridi* strain RCAM05007 obtained from long reads. The strain was isolated from the root nodule of *Cyamopsis tetragonoloba* (L.) Taub. plant inoculated with a soil sample from India. The assembly contains one chromosome and two megaplasmids totalling ~6.7 Mbp with 61.6% GC content.

## ANNOUNCEMENT

Guar [*Cyamopsis tetragonoloba* (L.) Taub.] is a key legume crop in arid areas of Asia. The seeds of this crop produce guar gum, a natural thickener used as a high-viscosity surfactant in various industries, including food, medicine, cosmetics, textiles, and energy (1, 2). As a legume, guar forms symbiotic relationships with rhizobia, which fix atmospheric nitrogen and provide essential nutrients to the plant. However, effective strains are needed to enhance plant growth, yield, and seed quality, and the *Ensifer aridi* RCAM05007 strain could contribute to these improvements (3). The strain *Ensifer aridi* RCAM05007 was isolated from the nodule of *C. tetragonoloba*. Guar seeds of the Kuban Yubileiny variety were scarified and sterilized in 98% H_2_SO_4_ for 30 min and then germinated in Petri dishes at 25°C in the dark for 3 days. Seedlings were transferred to sterile polypropylene pots with vermiculite, inoculated with soil suspension from Nagaur region (India), and grown for 30 days. Individual nodules were collected, sterilized for 1 min in 70% ethanol, rinsed, homogenized in sterile tap water, and plated on the YMSA medium [modified yeast extract mannitol agar (YMA) supplemented with 0.5% succinate] (4). Colonies appeared after 3 days of incubation at 28°C. The strain RCAM05007 was purified by repeated re-streaking on the medium. DNA without size selection was isolated using the DNeasy Blood & Tissue KIT (Qiagen, Germany) according to the manufacturer’s recommendations from a pure culture grown in R2A broth at 28°C for 48 h. The library was prepared with the SQK-LSK109 ligation sequencing kit and the EXP-NBD104 native barcoding expansion 1–12 kit (ONT, UK), skipping the DNA-shearing step and sequenced using a MinION sequencer with Flow Cell R.9.4.1 (ONT, UK). Basecalling was performed using Guppy (v 5.0.11) with the “high-accuracy” model (5). The sequencing resulted in 225,590 reads with an average read length of 6,238 bp. Quality control of the raw reads was provided by NanoStat (v 1.6.0) (6). The genome sequence was assembled using the Flye assembler (v 2.9), and default parameters were used for all software unless otherwise noted (7). Assembly statistics were provided by QUAST (v.5.0.2) (8). Three circular contigs assembled without rotation—one chromosome of 3,687,889 bp in length and two megaplasmids: one of 2,274,007 bp and another of 770,709 bp—were assembled with a total size of approximately 6.7 Mbp, a GC content of 61.6%, and a mean coverage of ~201×. A phylogenetic analysis based on 16S rRNA gene BLASTn and average nucleotide identity (ANI) comparison using the JSpeciesWS online service indicated that strain RCAM05007 belongs to the genus *Ensifer* and is closely related to the type strain of *E. aridi* LMR001T (NR_178817.1; GCF_002078505.1) exhibited 100% and 97.91% identity, respectively (9, 10). Assembly annotation using PGAP (v 6.4) resulted in 5,968 protein coding and 68 RNA genes (9 rRNAs, 55 tRNAs, 4 ncRNAs) (11).

## Data Availability

All data are available at GenBank under BioProject accession number PRJNA899814. The BioSample accession number is SAMN42486122.The raw MinION data can be found under the number SRR29845158, the assembly accession number for the chromosome is CP168397.1, and the accession numbers for the plasmids are CP168398.1 and CP168399.1.
